# Supplementary material for: Virtual Vibrational Analytics of Reduced Graphene Oxide
Source: Int J Mol Sci. 2022 Jun 23;23(13):6978. doi: 10.3390/ijms23136978 (PMC9266465; doi:10.3390/ijms23136978)
Supplement: Supplementary file 1 [file ijms-23-06978-s001.zip › ijms-1729543-supplementary.pdf]

Virtual Vibrational Analytix of Reduced Graphene Oxide

Elena F. Sheka, Nadezhda A. Popova

Institute of Physical Researches and Technology,
Peoples' Friendship University of Russia (RUDN University), 117198 Moscow, Russia

sheka@icp.ac.ru

SUPPORTING INFORMATION

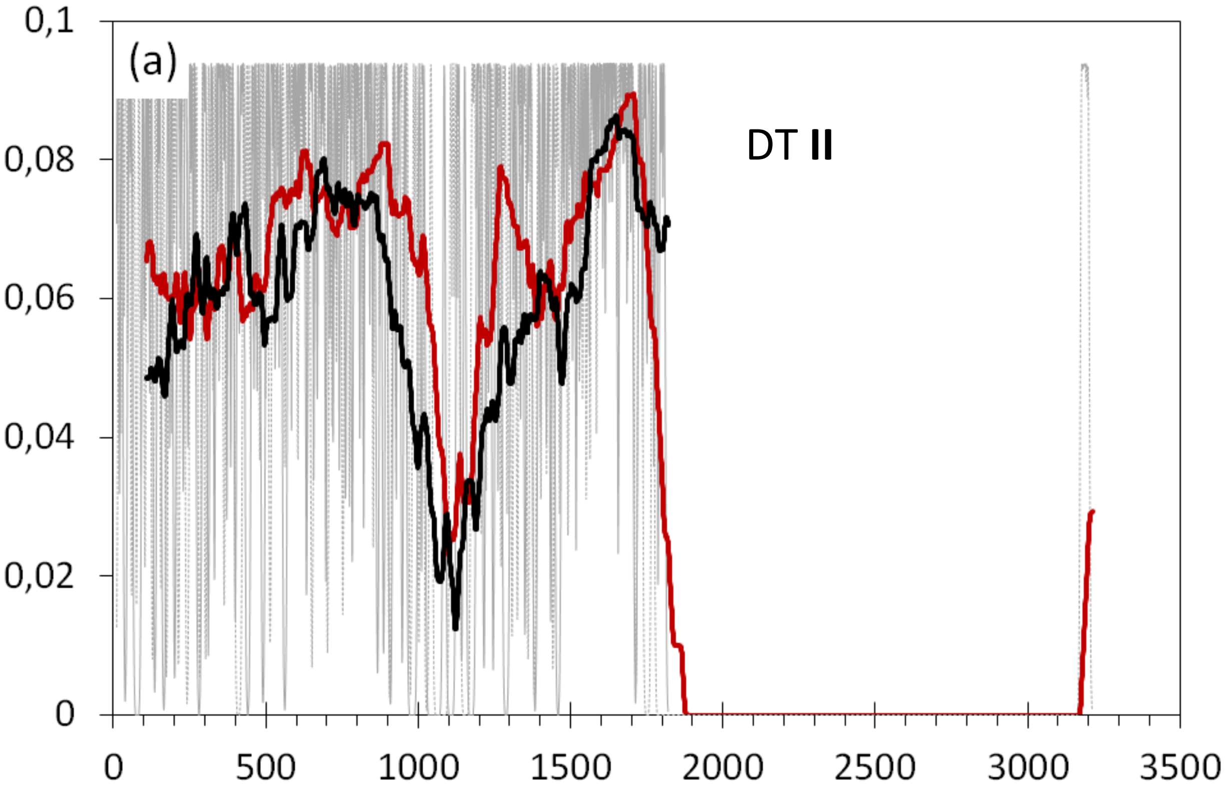

Figure S1. DOV spectra of DT **II** (red) and DT **I** (black). The DOVs trend lines correspond to 100-point linear filtration. UHF AM1 calculations.

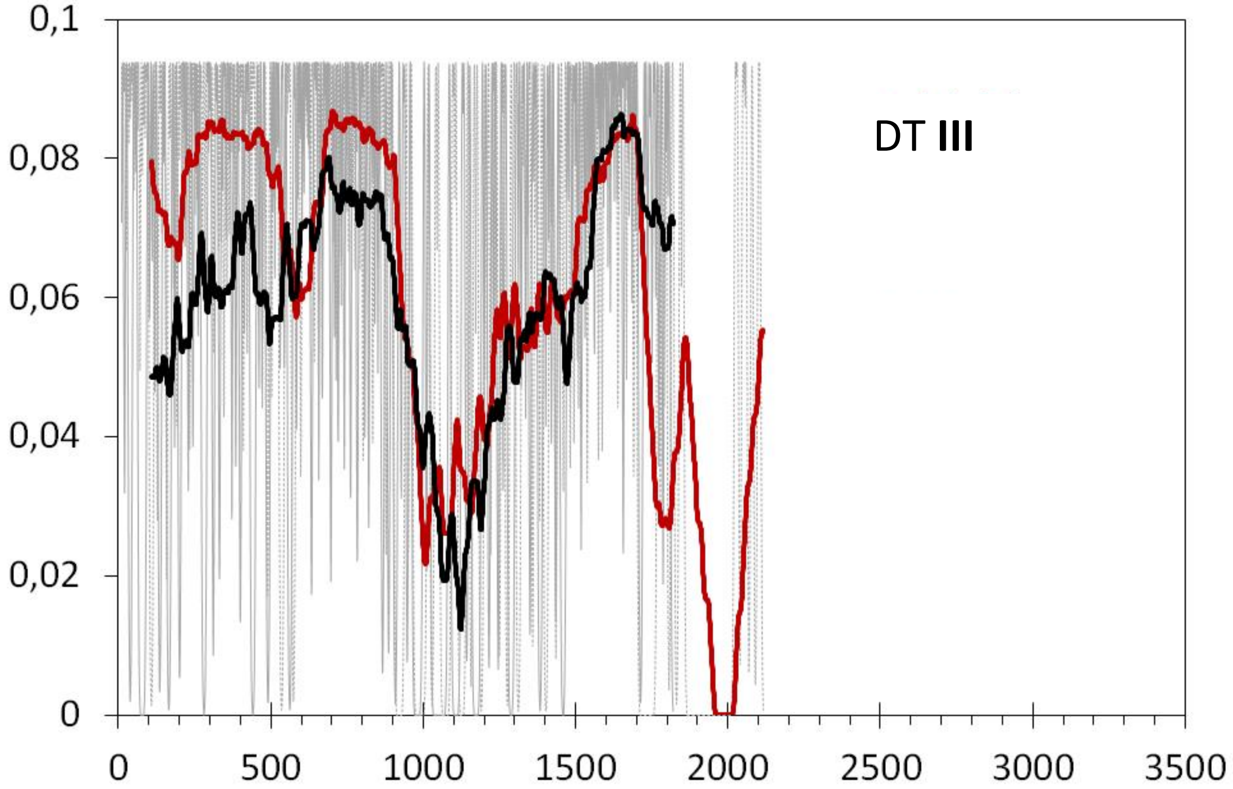

Figure S2. DOV spectra of DT **III** (red) and DT **I** (black). The DOVs trend lines correspond to 100-point linear filtration. UHF AM1 calculations.

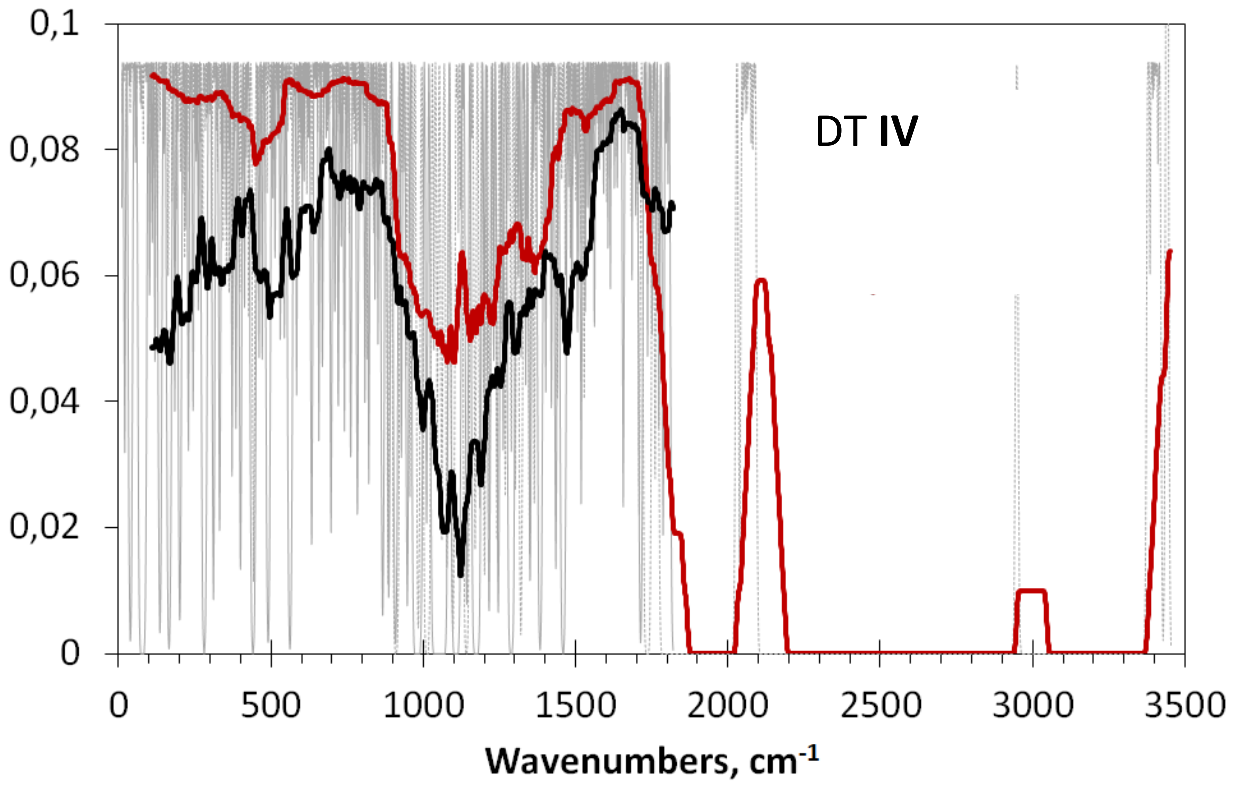

Figure S3. DOV spectra of DT **IV** (red) and DT **I** (black). The DOVs trend lines correspond to 100-point linear filtration. UHF AM1 calculations.

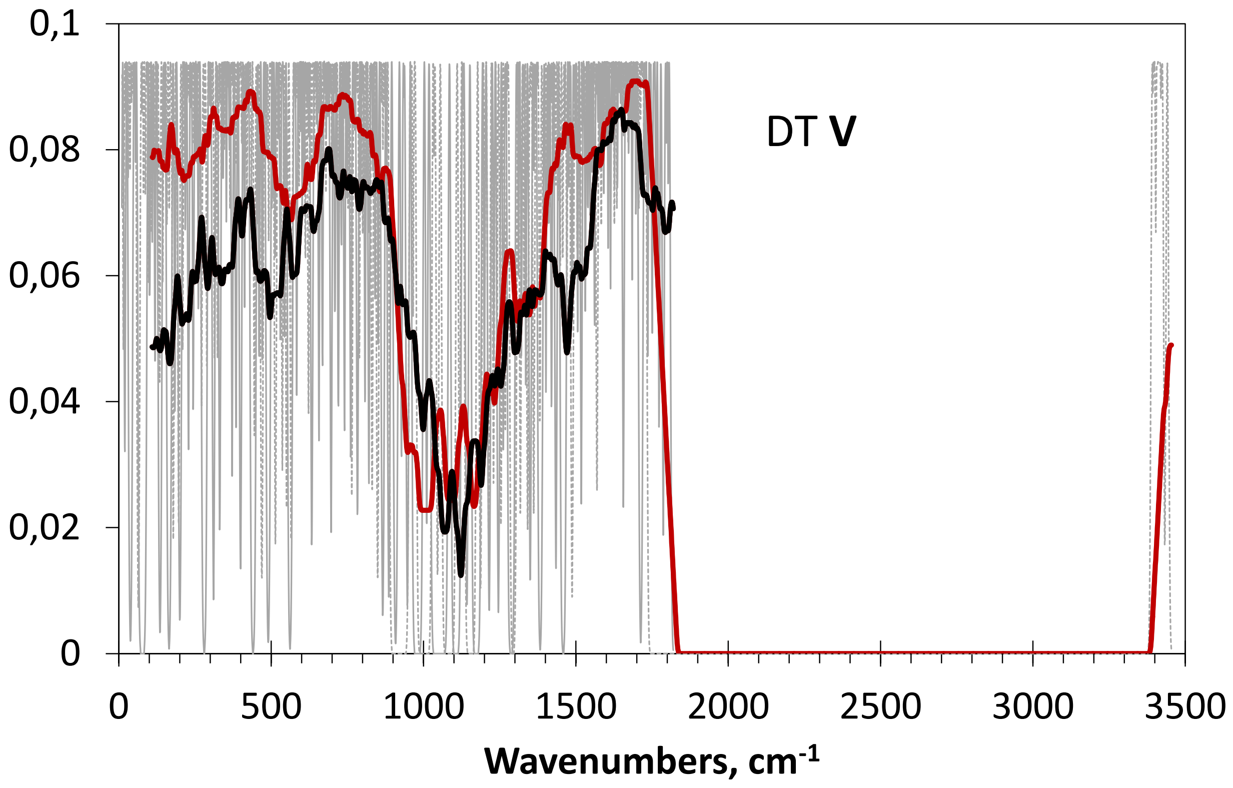

Figure S4. DOV spectra of DT **V** (red) and DT **I** (black). The DOVs trend lines correspond to 100-point linear filtration. UHF AM1 calculations.

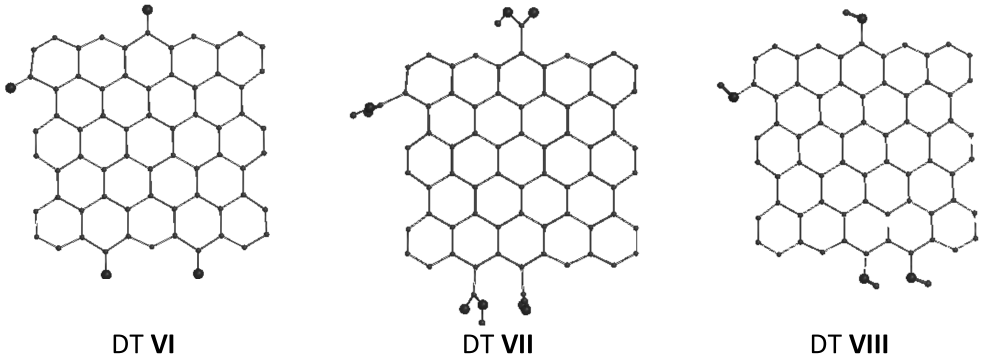

Figure S5. Equilibrated structures of DTs **VI-VIII**. UHF AM1 calculations.

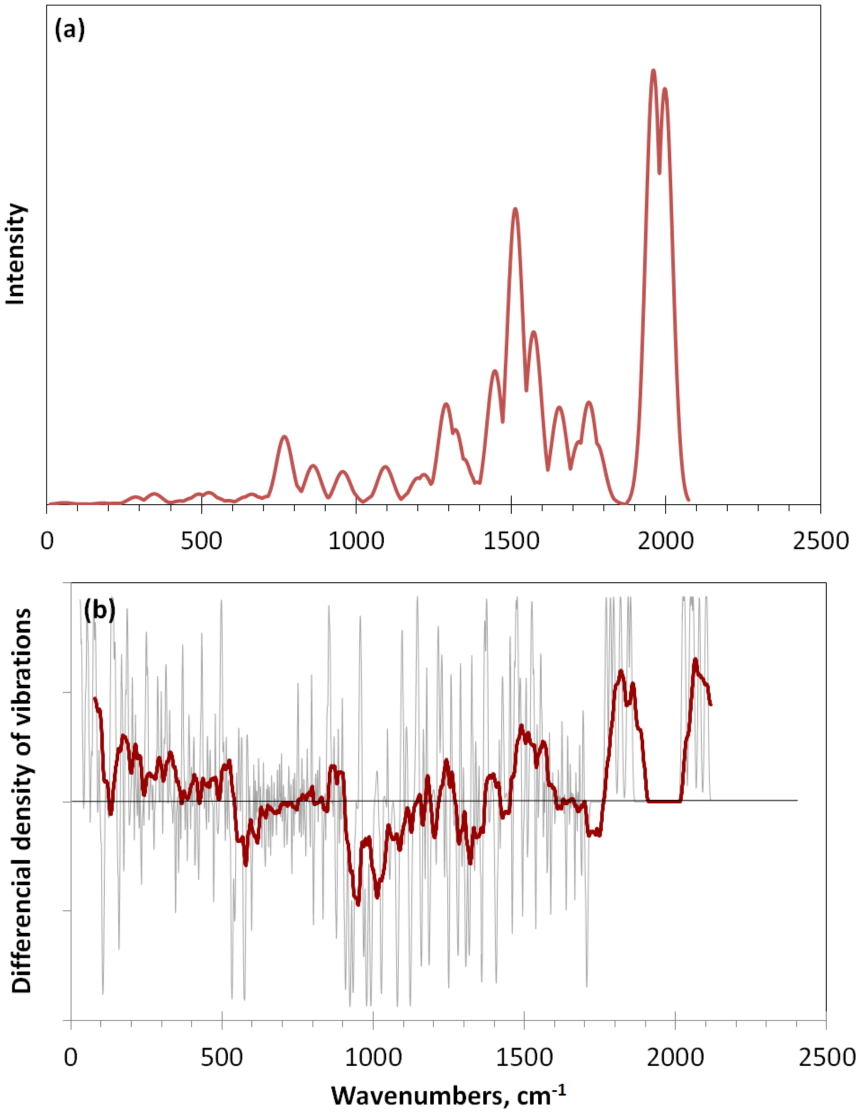

Figure S6. a. Virtual one-phonon IR spectrum of DT **III**. Gaussian convolution of the original stick bars performed with FWHM of 60 cm^{-1}. b. Differential DOV ΔV_3. Trend line corresponds to 50-point linear filtration. UHF AM1 calculations.

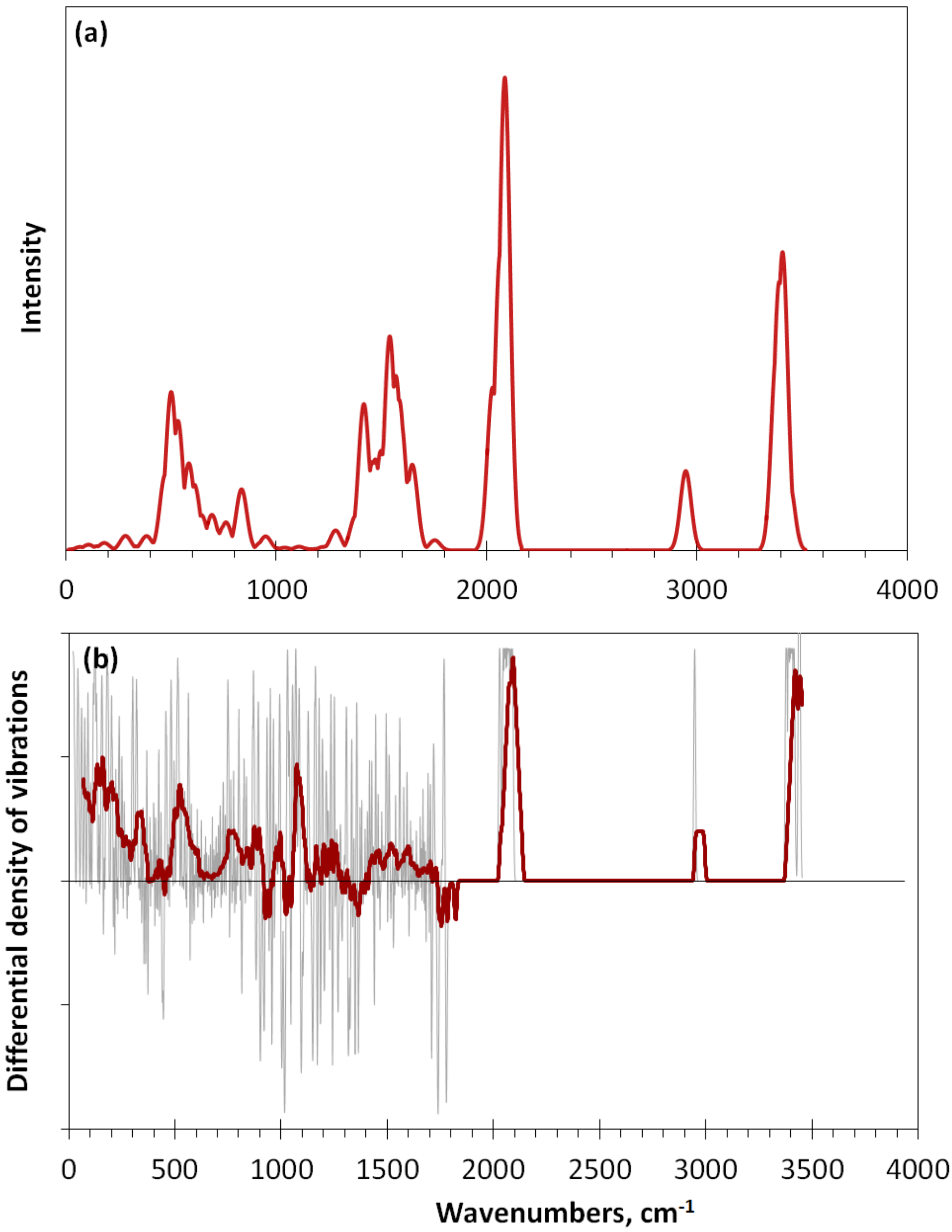

Figure S7. a. Virtual one-phonon IR spectrum of DT **IV**. Gaussian convolution of the original stick bars performed with FWHM of 60 cm^{-1}. b. Differential DOV ΔV_3. Trend line corresponds to 50-point linear filtration. UHF AM1 calculations.

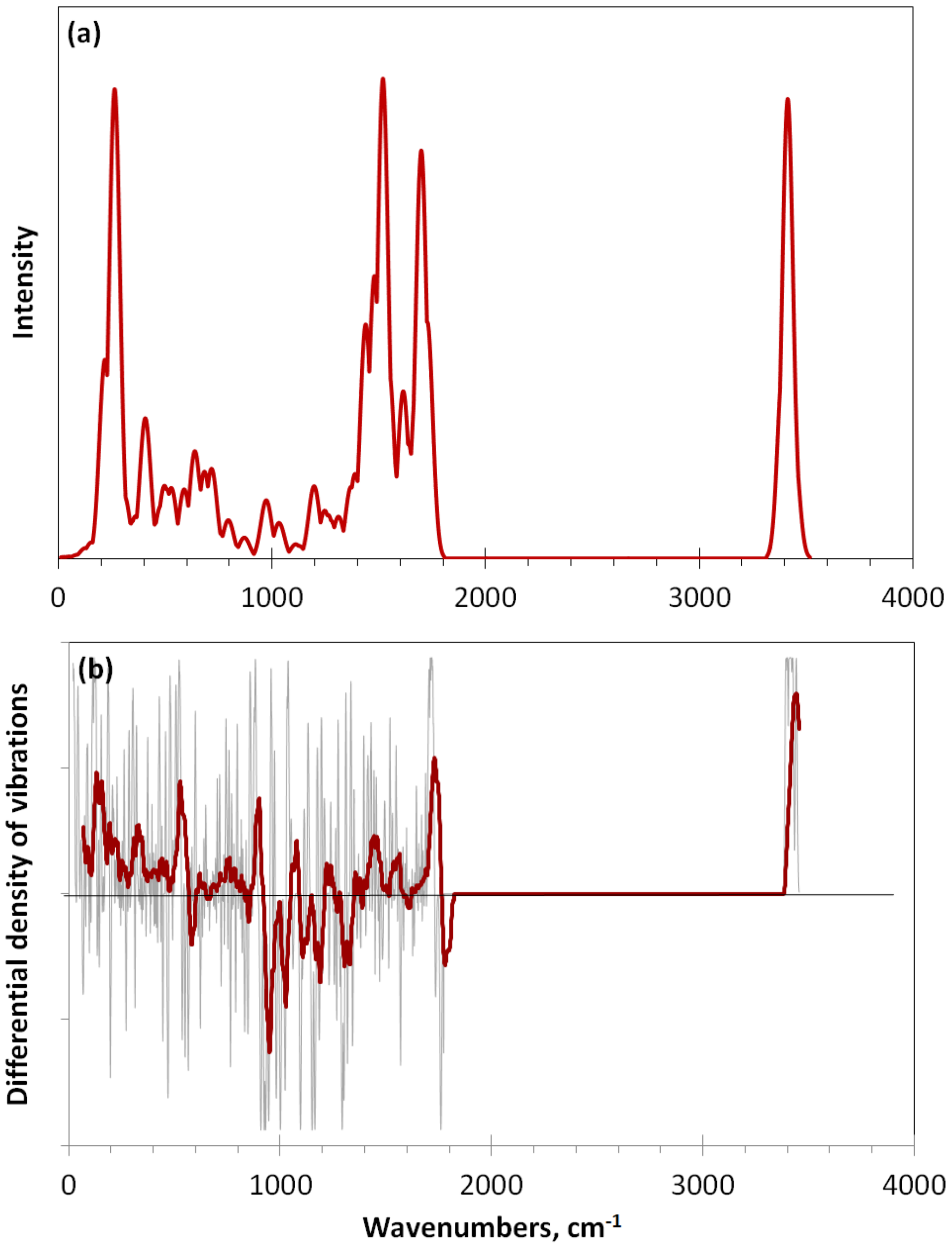

Figure S8. a. Virtual one-phonon IR spectrum of DT **V**. Gaussian convolution of the original stick bars performed with FWHM of 60 cm^{-1}. b. Differential DOV ΔV_3. Trend line corresponds to 50-point linear filtration. UHF AM1 calculations.
